# Three-dimensional ultrastructure analysis of organelles in injured motor neuron

**DOI:** 10.1007/s12565-023-00720-y

**Published:** 2023-04-18

**Authors:** Hiromi Tamada

**Affiliations:** 1 Functional Anatomy and Neuroscience, Nagoya University Graduate School of Medicine, 65 Tsurumai-cho, Showa-Ku, Nagoya, Aichi 466-8550 Japan; 2 Anatomy, Graduate School of Medicines, University of Fukui, Matsuokashimoaizuki, Eiheiji-Cho, Yoshida-Gun, Fukui 910-1193 Japan

**Keywords:** Organelle, Mitochondria, Three-dimensional ultrastructure, Injured motor neuron, FIB/SEM

## Abstract

Morphological analysis of organelles is one of the important clues for understanding the cellular conditions and mechanisms occurring in cells. In particular, nanoscale information within crowded intracellular organelles of tissues provide more direct implications when compared to analyses of cells in culture or isolation. However, there are some difficulties in detecting individual shape using light microscopy, including super-resolution microscopy. Transmission electron microscopy (TEM), wherein the ultrastructure can be imaged at the membrane level, cannot determine the whole structure, and analyze it quantitatively. Volume EM, such as focused ion beam/scanning electron microscopy (FIB/SEM), can be a powerful tool to explore the details of three-dimensional ultrastructures even within a certain volume, and to measure several parameters from them. In this review, the advantages of FIB/SEM analysis in organelle studies are highlighted along with the introduction of mitochondrial analysis in injured motor neurons. This would aid in understanding the morphological details of mitochondria, especially those distributed in the cell bodies as well as in the axon initial segment (AIS) in mouse tissues. These regions have not been explored thus far due to the difficulties encountered in accessing their images by conditional microscopies. Some mechanisms of nerve regeneration have also been discussed with reference to the obtained findings. Finally, future perspectives on FIB/SEM are introduced. The combination of biochemical and genetic understanding of organelle structures and a nanoscale understanding of their three-dimensional distribution and morphology will help to match achievements in genomics and structural biology.

**Supplementary Information:**

The online version contains supplementary material available at 10.1007/s12565-023-00720-y.

## 3D morphological analysis for organelle in injured motor neurons

When cells encounter stress, the organelles respond drastically and alter their shapes to maintain cellular homeostasis. Mitochondria, which are particularly important in the nervous system for energy production, Ca^2+^ regulation, and maintenance of plasma membrane potential, have been studied extensively in neurological and nerve injury models (Knott et al. 2008a; Berman et al. 2009; Bilsland et al. 2010; Sheng and Cai 2012; Sleigh et al. 2019; Han et al. 2020; Licht-Mayer et al. 2020; Collier et al. 2023).

Mitochondrial dynamics, that undergo a continuous cycle of fission and fusion, maintain their own homeostasis, and regulate cellular homeostasis as well. Regulatory proteins are now well characterized and their ablation leads to neurodegenerative diseases (Giacomello et al. 2020; Cheng et al. 2022). In particular, mitochondrial fission with dynamin-related protein 1 (Drp1) is one of the representative factors for mitochondria to be transported effectively and accelerate nerve regeneration after injury (Kiryu-Seo et al. 2016; Pozo Devoto et al. 2022).

**Fig. 1:**
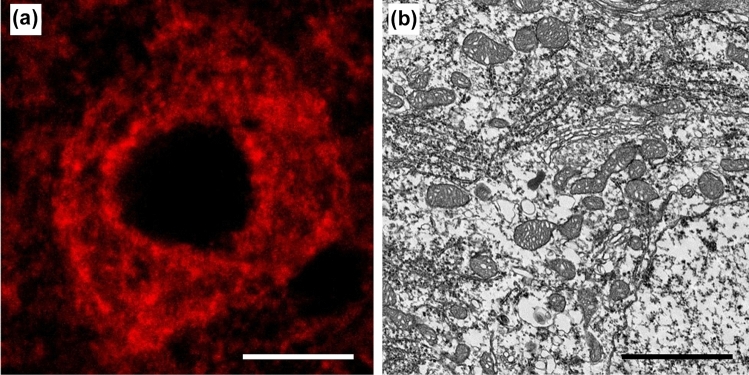
The difficulty in observations of mitochondria in cell bodies with conditional microscopy **a** The immunohistochemistry with cytochrome c (CytC) for the neuronal cell bodies in mouse hypoglossal nucleus stained the whole cytoplasm and it was impossible to detect each mitochondrion because of their sizes and richness. **b**The transmission electron microscopy showed each mitochondrion, but the whole structure couldn’t be understood. Scale bar **a** 10 µm; **b** 2 µm (Images from Tamada et al. (2017) J Comp Neurol)

Sciatic nerve and hypoglossal nerve transection models are frequently used as regenerative models after injury (Nakagomi et al. 2003). Since the condition without isolated cells and cultured cells is relatively close to in vivo situation, studying the response in this model might help explain some regenerative mechanisms. However, the mitochondria within neuronal cell bodies display a rich distribution and possess innumerable tiny structures. These make their individual detection almost impossible due to the limited resolution of light microscopy (Fig. 1a). Each mitochondrion can be detected using transmission electron microscopy (TEM), but it is still difficult to understand the whole structure and analyse it quantitatively (Fig. 1b). Then, Focused ion beam/scanning electron microscopy (FIB/SEM) is a powerful tool to solve this problem.

**Fig. 2:**
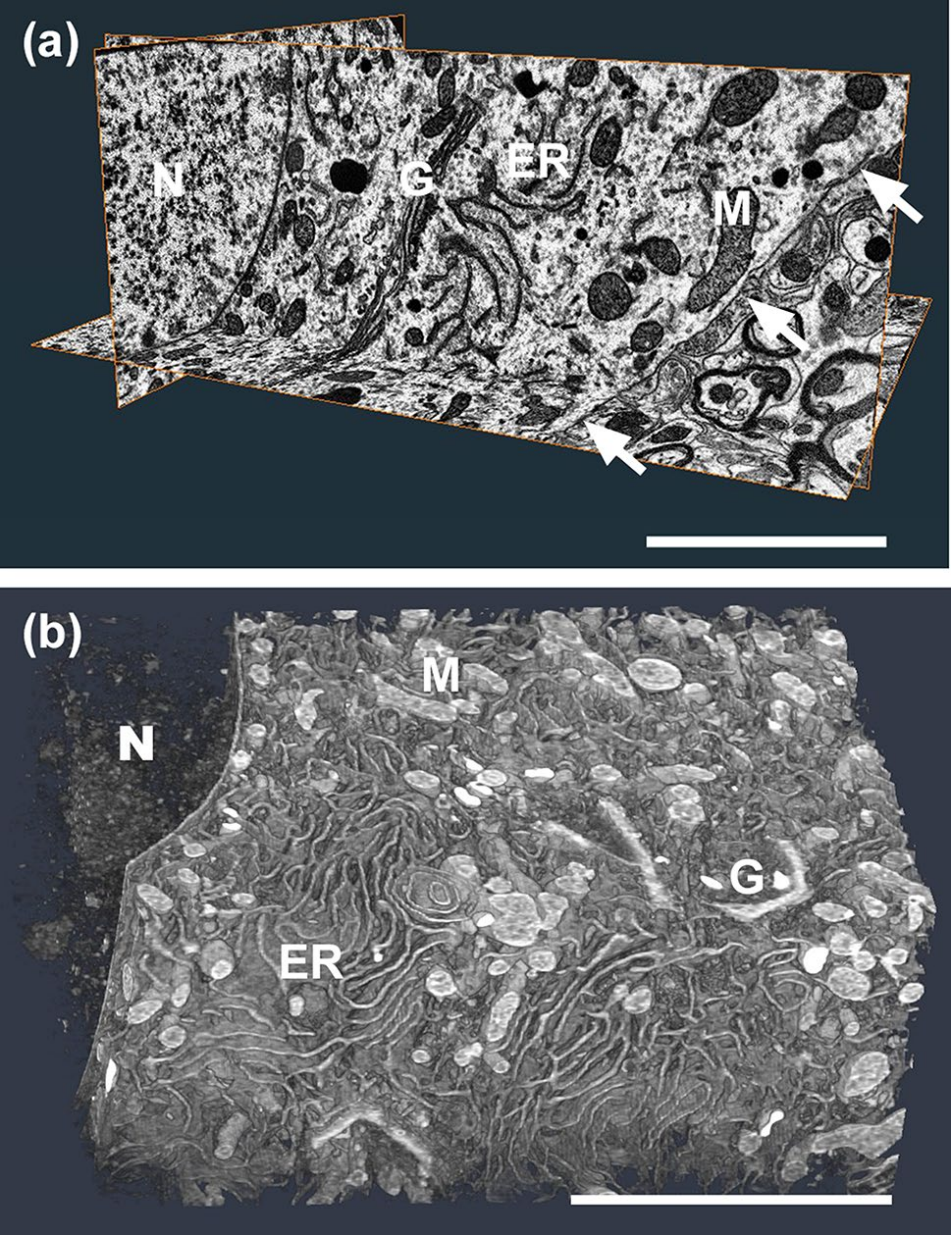
FIB/SEM images in soma **a** The representative x, y, z EM images could be obtained with serial images from FIB/SEM. **b** The three-dimensional ultrastructures of organelle in neurons could be understood with the FIB/SEM images. N: nucleus, ER: endoplasmic reticulum, G: Golgi body, M: Mitochondria, arrows: cell membrane Scale bar 5 µm

The FIB/SEM is a novel EM technique for obtaining volume EM images (Knott et al. 2008b; Merchán-Pérez et al. 2009; Ohta et al. 2012; Narayan and Subramaniam 2015). In addition to FIB/SEM, serial block face-scanning electron microscopy (SBF-SEM) (Denk and Horstmann 2004; Wilke et al. 2013) and automated tape-collecting ultramicrotome SEM (ATUM-SEM) (Hayworth et al. 2014; Morgan et al. 2016) are used. Under these methods, the x, y, z-axis images can be obtained by acquiring tremendous number of serial EM images and stacking them (Fig. 2a). This makes it possible to know three-dimensional ultrastructure even for very tine organelle in cells (Fig. 2b). They possess different advantages depending on the process of creating the observing surfaces. One of the key advantages of FIB/SEM is that very small z-pitches (~ 10 nm) intervals are obtained by milling gallium ion beams, which is not possible with other methods that uses diamond knives. This allows the observation of small organelles which tend to be distributed close to each other and their membrane-contact sites.

## 3D ultrastructure of mitochondria in cell bodies

**Fig. 3:**
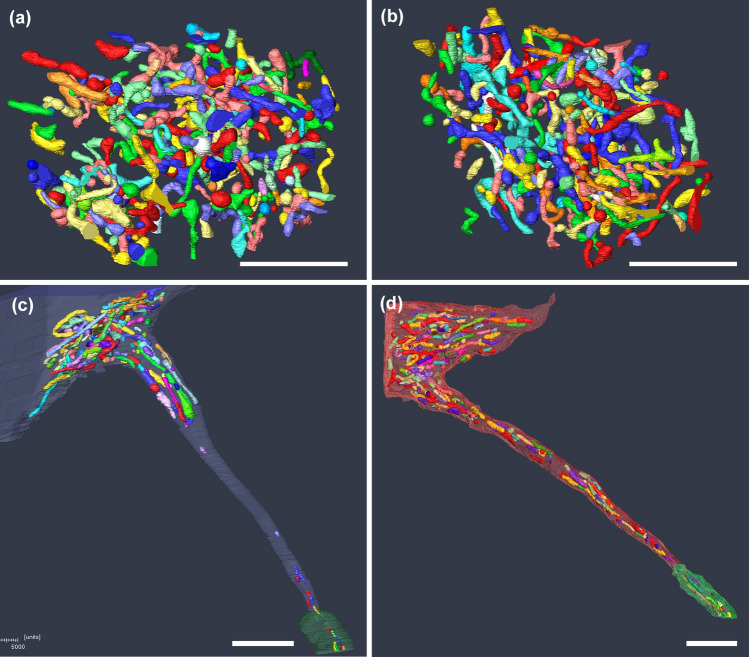
FIB/SEM analysis of mitochondria in soma and AIS **a** Each mitochondrion in soma of healthy motor neurons was reconstructed in different colours. Tremendous number of mitochondria showing round and tubular shapes were distributed. **b** Reconstructed mitochondria in soma of motor neuron at one week after injury showed almost the same features as that in healthy ones. **c** The AIS in healthy motor neurons didn’t show any mitochondria distribution, although the cell bodies, the axon hillock and the myelinated region did. (blue: cell membrane including AIS region, green: myelin sheath) **d** At one week after injury, mitochondria distribution could be observed even in the AIS. Their shapes and sizes of mitochondria here varied as the same as that distributed in the soma, the axon hillock, and the myelinated region. (red: cell membrane including AIS region, green: myelin sheath) Scale bar 5 µm [Images from Tamada et al. (2017) J Comp Neurol and Tamada et al. (2021) J Comp Neurol]

The FIB/SEM analysis revealed a high degree of morphological variability of mitochondria in the soma, that is, length, width, and roundness (Fig. 3a) (Tamada et al. 2017). One of the unexpected findings in the soma after injury was that the mitochondrial sizes did not change drastically (Fig. 3b), although it has been well established that mitochondrial fission is accelerated after injury and transported into the axon tips to provide ATP sources for nerve regeneration (Cho et al. 2009; Wang and Schwarz 2009; Kiryu-Seo et al. 2010; Chamberlain and Sheng 2019). It is possible that the morphological response of mitochondria in axons is not necessarily the same as that in soma, because axonal mitochondria exist in unique environments and possess different properties than somatic mitochondria (Hollenbeck and Saxton 2005; Pathak et al. 2013). In addition, since the mitochondrial transport speed is not very fast and it is assumed to take several days to convey mitochondria from the soma to the distal axon part (Sheng 2017), mitochondrial fission regulation may occur not only in the soma but also in the distal axonal part.

As described above, mitochondria repeat fission and fusion for maintaining their homeostasis and cellular homeostasis. The regulating proteins have been explored well and dynamin-related protein 1 (Drp1) is one of the crucial proteins for fission (Ishihara et al. 2009; Kageyama et al. 2011). The analysis of injury-induced Drp1 knockout (KO) mice with FIB/SEM demonstrated alternation processes from healthy shapes to degenerative structures (Tamada et al. 2017). In a previous study, the mitochondrial fission protein Drp1 was found to be crucial for nerve regeneration, and giant mitochondria were detected after injury in the same Drp1 KO mouse (Kiryu-Seo et al. 2016). However, the question of how these structures were formed still remains unsolved. The FIB/SEM analysis in Drp1 KO mice showed spherical mitochondria together with single or multiple long and thin processes at one week after injury (Supp Fig. a) (Tamada et al. 2017). In some cases, one or more spherical mitochondria are connected by tubular mitochondria. Furthermore, the tubular processes of mitochondria disappeared, showing an extremely large round shape, two weeks after injury (Supp Fig. b). This indicated that the mitochondria did not merely swell uniformly, but initially formed long tubules by connecting nearby mitochondria and subsequently enlarged to form a balloon structure. Based on quantitative analysis, their volume was 10-fold more than their volume in non-injured motor neurons (3.0 × 10^8^ nm^3^ in non-injured vs 1.0 × 10^9^ nm^3^ in KO mice). This means that under normal conditions, less than 10% of the cytoplasmic volume was occupied by mitochondria, but 50% was occupied at two weeks after injury in the Drp1 inhibited model.

With FIB/SEM, details of the inside of the mitochondria and cristae structures can also be observed. These extremely large spheroid mitochondria were finally degraded with the collapse of the interior and mitophagy-like processes accompanied by lysosomes (Supp Fig. c, d) (Tamada et al. 2017). The degradation of mitochondria from the inside indicated that not only the inhibition of mitochondrial transport to axonal tips but also the collapse of mitochondrial quality control occurred in this Drp1 KO injury model. Usually, mitochondrial homeostasis is maintained by the clearance of damaged mitochondria. In this process, Drp1-dependent fragmentation is crucial (Giacomello et al. 2020). In the Drp1 KO injury model, natural mitophagy accompanied by healthy fission did not occur, and the whole degenerated spherical mitochondria were wrapped with isolation membranes (Tamada et al. 2017).

FIB/SEM analysis provides information about mitochondrial membrane dynamics, which are linked to several common diseases (Giacomello et al. 2020). These results would also accelerate the understanding of mitochondrial pathology and explore their therapies.

## 3D ultrastructure of mitochondria in AIS

The axon initial segment (AIS), between the end of the axon hillock and the beginning of the first myelin sheath, is an anatomically and physiologically crucial point when mitochondria are transported from somata to axons. Since AIS is the axonal domain responsible for action potential initiation, internal structures and membrane circumstances are characteristic (Nelson and Jenkins 2017; Leterrier 2018). The distribution of mitochondria in AIS is obscure compared to that in axons (Zhang et al 2010; Ohno et al 2011; Cheng et al 2022). This may be because it is extremely difficult to access the AIS, which are small and short regions embedded in neuronal tissues, rendering it impossible to isolate them clearly. Next, we demonstrated the mitochondrial distribution in AIS using FIB/SEM.

Surprisingly, normal AIS possessed almost no mitochondrial distribution, although a large number of mitochondria could be detected in the cell bodies, axon hillock, and myelinated axons (Fig. 3c, Fig, 4) (Tamada et al. 2021). At present, although it is unclear why mitochondria are not distributed only in the AIS region, we speculate two possibilities: (1) the transport velocity along the AIS is so rapid that they cannot be captured, (2) only the motile mitochondria are distributed in the AIS, although some mitochondria move persistently in axons and some are anchored or stationary (Morris and Hollenbeck 1993; Pilling et al. 2006; Ohno et al. 2011).

**Fig. 4:**
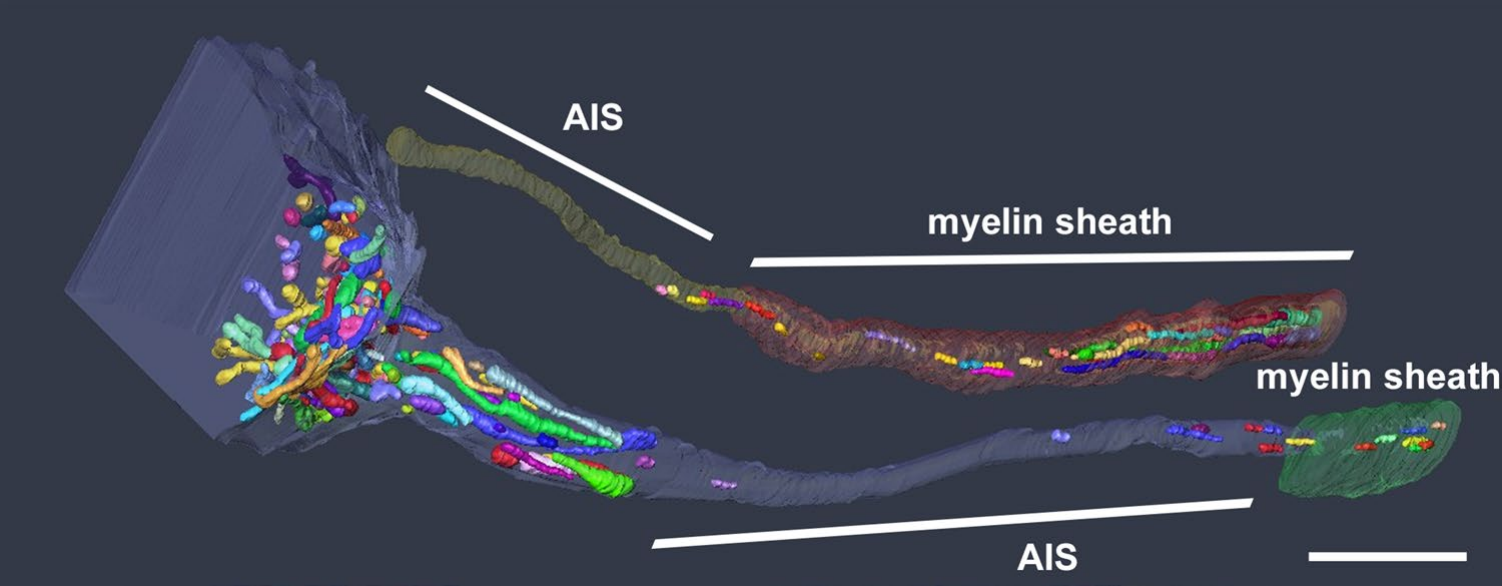
The details of mitochondria distribution in the healthy AIS. In addition to the AIS shown in Fig. 3c, the other AIS and long myelinated areas are shown. Again, no mitochondria were observed in the AIS region. Scale bar 5 µm

Frequently, the AIS and the nodes of Ranvier are described as similar structures because they share a common ion channel and a common cytoskeleton comprised of βIV spectrin and ankyrin G (AnkG) (Kordeli et al. 1995; Berghs et al. 2000; Chang and Rasband 2013; Zollinger et al. 2015). According to some studies, there is a variation in the distribution of mitochondria within the node of Ranvier. For example, some nodes do not possess mitochondria or some have a few mitochondria (Berthold et al. 1993; Fabricius et al. 1993; Edgar et al. 2008; Ohno et al. 2011; Perkins & Ellisman 2011). Even in the case of mitochondria existences, their size indicated motile mitochondria with short shapes, although the stationary mitochondria with long shapes were not distributed (Ohno et al. 2011). These results are consistent with the hypothesis that mitochondria does not accumulate in the normal AIS because the stationary mitochondria are not likely to exist. Although the results of nodes of Ranvier tend to vary depending on the areas that come across due to their short lengths, the continuous observation of whole AIS structures by FIB/SEM could show more obvious trends.

**Fig. 5:**
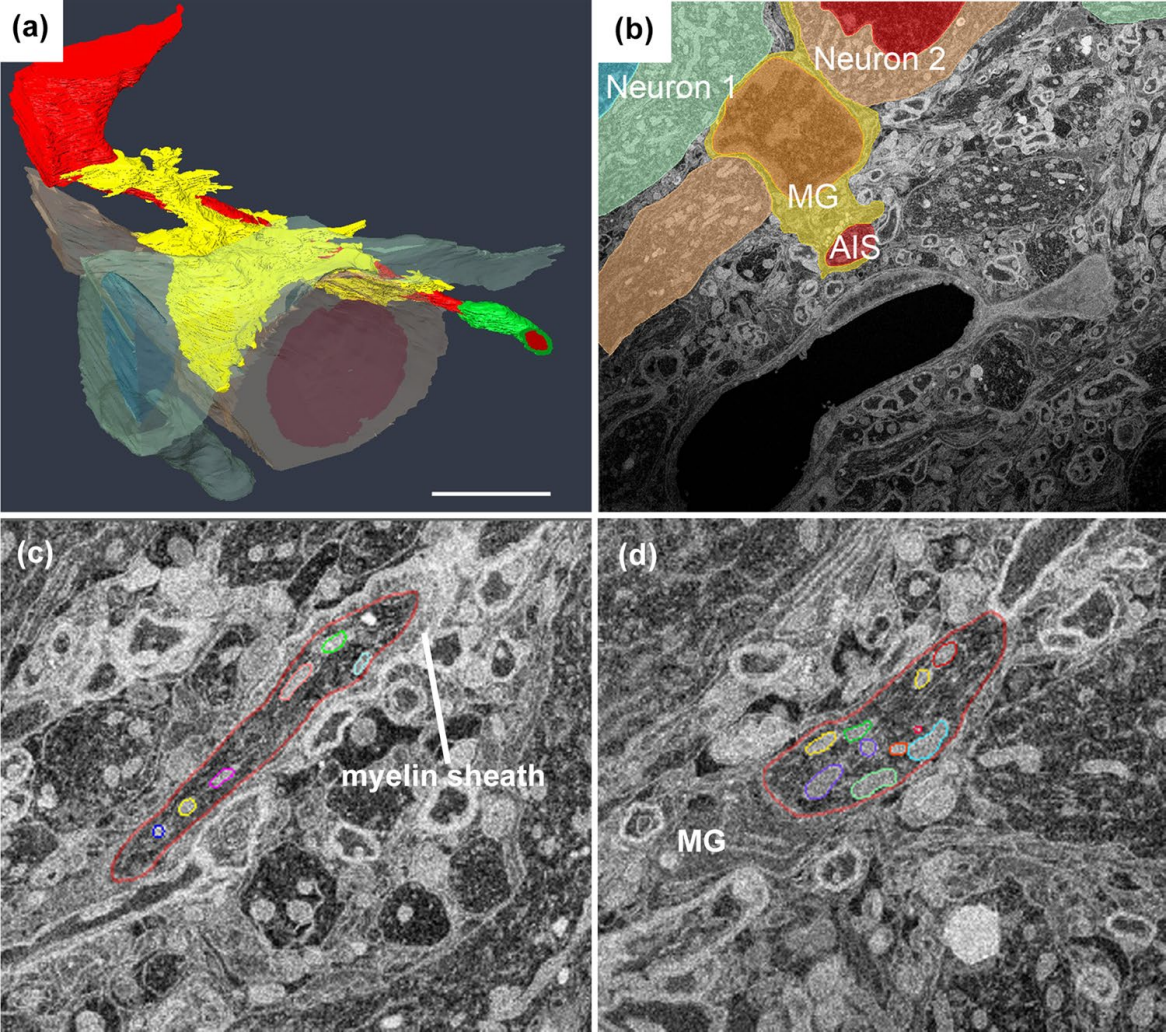
3D reconstructed images of microglia around AIS **a** 3D reconstructed images show the details of the surrounding microglia. (Red: neurons with AIS, green: myelin sheath, yellow: microglia, blue and orange: the other neurons which the same microglia attaches). **b** Representative one-slice SEM images for Fig. 5a showing one AIS (red), two neurons (green and pink), and one microglia (yellow) attached to them. There are no other structures between microglia and other elements, indicating that microglia make direct membrane contact with AIS and neuronal cell bodies. **c** and **d** Representative serial SEM images around AIS of Fig. 5a, b. AIS is surrounded in red line and each colours in the AIS are showing mitochondria. Scale bar **a** 5 µm (Images from Tamada et al. (2021) J Comp Neurol)

Meanwhile, after axon injury, a tremendous number and varying length of mitochondria could be observed even in the AIS, myelinated area, axon hillock, and somata (Figs. 3d,  5c, d) (Tamada et al. 2021). At the same time, microglia adhesion around AIS was also explored in the same adhesion manner as that around cell bodies (Fig. 5a, b). These results indicated that the intracellular and extracellular conditions of AIS and somata are likely to become homogenous. However, mitochondrial influx in AIS could also be detected when the crush mild injury model was adopted to maintain AnkG expression (Tamada et al. 2021), which is the main scaffolding protein for AIS assembly and maintenance (Buffington and Rasband 2011). The finding indicates that this phenomenon of mitochondria is not dependent on cytoskeletal depletion.

Thus, the interaction between microglia and mitochondrial localisation could be considered. Although microglial contacts to the AIS have been reported during development and for maintaining axon conditions such as in multiple sclerosis, traumatic brain injury, and acute neuroinflammatory conditions (Baalman et al. 2015; Clark et al. 2016; Benusa et al. 2017; Benusa and Lafrenaye 2020; Gallo et al. 2022), the functional details and consistent understanding have not yet been explored. Benusa et al. (2017) suggested that the expression of microglia around the AIS increases the intracellular Ca^2+^ concentration in the AIS and results in the disruption of AnkG expression secondary to calpain, which is regulated by Ca^2+^ (Schafer et al. 2009; Benned-Jensen et al. 2016). Mitochondria possess a Ca^2+^ regulatory system, and the mitochondrial transport is attenuated in the axon when cytosolic Ca^2+^ levels are elevated (Macaskill et al. 2009; Wang and Schwarz 2009; Zhang et al. 2010; Ohno et al. 2011; Saxton and Hollenbeck 2012; Kontou et al. 2021; Kole et al. 2022). With all things considered, some regulatory systems coordinated with microglia, mitochondria, and Ca^2+^ might occur in AIS in this injury model. Although the details of the function of microglia in AIS have not yet been explored, the microglial attachment does not seem to have a strong and direct influence on AIS collapse with AnkG disruption (Clark et al. 2016; Benusa et al. 2017). Instead, they might mildly maintain AIS circumstances.

Recently, AIS-related mechanisms for nerve regeneration and neurodegenerative disorders have been studied (Kiryu-Seo et al. 2022; Teliska et al. 2022; Tjiang and Zempel 2022). Parallel analysis with FIB/SEM for intra- and extracellular milieu in three dimensions can provide a new comprehensive research target and lead to a new therapy.

## Future issues of organelle analysis with FIB/SEM

This review focuses on mitochondrial structures. Furthermore, the endoplasmic reticulum (ER) is also an important factor in regulating cell homeostasis (Westrate et al. 2015; Hetz and Saxena 2017; Marciniak et al. 2022) and the membrane attachment between mitochondria and ER (mitochondria-associated membrane: MAM) (Kornmann et al. 2009; Csordás et al. 2010; Friedman et al. 2011; Prinz et al. 2020) is also a hot topic in neurological diseases (Hedskog et al. 2013; Paillusson et al. 2016; Watanabe et al. 2016; Area-Gomez et al. 2018; Kim et al. 2022). Apart from MAM structures, mitochondria possess a variety of signalling pathways in cells, such as mitochondria-derived vesicles (MDV) (Sugiura et al. 2014; Matheoud et al. 2016; König et al. 2021). For these tiny structures, FIB/SEM analysis is required. FIB/SEM allows us to approach these structures in mammalian tissues, although these studies have been well-explored in yeast and *Drosophila* so far. Recently, it was reported that the fission point, where Drp1 also accumulates, determines the mitochondrial fate and functions after fission (Kleele et al. 2021; Ul Fatima and Ananthanarayanan 2023). It is also possible to determine the details of the location and attachment manner of the MAM structure in mitochondria with FIB/SEM. As such, the FIB/SEM analysis contributes to providing novel findings and exploring new study fields to understand the physiological meanings by showing organelle structures and interactions with surrounding elements at the ultrastructural level, which has never been seen before.

However, there are some technical challenges to FIB/SEM analysis. First, to perform integral analysis spatially and temporally, some attempts for new volume correlative light-electron microscopy (CLEM) have been challenged (Hayashi et al. 2023) with improved accuracy, throughput, and accessibility. These include new genetically labelling systems such as GFP and APEX (a soybean ascorbate peroxidase) (Martell et al. 2012; Okayama et al. 2014; Lam et al. 2015; Thomas et al. 2019), with arrangements of natural fiducial marks (Maclachlan et al. 2018) or artificial ones (Maco et al. 2014), developing the workflow of image acquisition by devices and application modification (Loginov et al 2022), and so on. Although the CLEM itself is the classic attempt to observe the same structures detected with light microscopy and electron microscopy, which could integrate physiological and ultrastructural data (Armer et al. 2009; Lidke and Lidke 2012; Maco et al. 2014; Blazquez-Llorca et al. 2015; Karreman et al 2016), the state-of-the-art CLEM is used to visualise the nanoscale relationship of specific proteins in the context of the global cellular ultrastructure (Hoffman et al. 2020), to link live-imaging data to high-resolution ultrastructural detail in three dimensions (Loginov et al. 2022) along with the development of other advanced technologies.

Second, sample preparation is also a topic to be discussed (Korogod et al. 2015; Tamada et al. 2020). The simulation model (Tønnesen et al. 2014) and computation geometry analysis (Kashiwagi et al. 2019) of spines with actual values of morphological data have been performed well, accompanied by the development of super-resolution microscopy. A variety of spines also affect their functional properties (Ofer et al. 2021) and consequently affect brain functions such as synaptic plasticity, learning, and memory. Therefore, more precise observations of native structures are required to obtain correct interpretations, and FIB/SEM analysis can be a suitable strategy for this analysis. However, since long ago, EM samples have been called into question because strong aldehydes are used for sample preparation that might convert native structures (Karlsson and Schultz 1965). Therefore, high-pressure cryo-fixation without aldehyde fixatives, which has been known also as the freeze-substitution (van Harreveld et al 1965), was performed in mouse brain tissues to maintain a more native structure (Tamada et al 2020). In the fixation, through the low-temperature embedding process after freezing, the sample in epoxy resin can be obtained as normal EM sample. The study suggested that some tiny structures, such as spine necks, tended to be swollen by conditional chemical fixation (Tamada et al. 2020). Because spine shape critically depends on the arrangement of actin, which is easily influenced by exposure to aldehydes (Honkura et al. 2008), this might contribute to the differences between cryo- and chemical-fixed tissue. The value of FIB/SEM required in the future will depend on the extent to which it reveals the native ultrastructure and whether these cells are representative of the physiological state (Hoffman et al. 2020). Furthermore, also for the study field of organelle, the influence of sample preparation might be one of the discussion points (Möbius et al. 2010). In this situation, more accurate methods, such as cryo-FIB/SEM (Schertel et al. 2013; Zachs et al. 2020), may be required to remove any possible structural changes. Because the present cryo-fixation has some limits technically, including difficulties for obtaining high-quality samples constantly which is strongly depending on quickness of preparation (Ohno et al. 2010) and limitations of effective sample depth, more technical improvement is required to obtain volume FIB/SEM images like the whole brain.

Third, the development of an auto-segmentation system using image processing and deep learning (DL) is a noteworthy field of study. The image segmentation is required to reconstruct the three-dimensional images from serial EM images. At present, the manual segmentation is broadly performed and is a very time- and labour-consuming task. Thus, the automated segmentation method is in high demand accompanied by an improved ability to acquire larger datasets by FIB/SEM, which exceeds the capacity of manual annotation. The main strategy for auto-segmentation is a three-dimensional convolutional neural network (CNN) architecture based on a three-dimensional U-Net (Çiçek et al. 2016; Falk et al. 2019). Some developing tools have been released by an open source, such as a repository providing large quantities of reliable data, codes, and trained models (Heinrich et al. 2021), a new pipeline to train a CNN effectively (Gallusser et al. 2023), improved DL platforms (Suga et al. 2021), and so on. However, more challenges remain in an ongoing effort to reduce human labour for the generation of training data, proofreading predictions, and reducing computation costs. Because innovation in this field has largely been driven by connectomics focusing on segmentation of cell and synaptic junctions (Kreshuk et al. 2011; Dorkenwald et al. 2017; Januszewski et al. 2018), further studies, especially on organelles, are awaited hereafter.

## Conclusion

With the arrival of new EM methods, we were able to gather three-dimensional ultrastructures even in tissue samples. This could accelerate a more direct approach for understanding biological questions. In particular, the ability of FIB/SEM to image cells and tissues at several-nanometre resolution over volumes as large as several tens of micrometers is an ideal tool. By solving some technical issues and combining several microscopic technologies, novel findings can be understood in the future.

## Supplementary Information

Below is the link to the electronic supplementary material.

FIB/SEM analysis of mitochondrial in injured neurons of Drp1 KO mouse (a) Each mitochondrion in soma of injured motor neurons was reconstructed in different colours. At one week after injury, some swollen mitochondria with processes are observed. (b) At two weeks after injury, extremely large round mitochondria without processes are detected. (c) Mitophagy-like structures with lysosomes are observed at two weeks after injury (green: mitochondria, pink: isolation membranes, purple: lysosome). (d) The representative SEM image for (c). The inner structures of mitochondria are also collapsed. Scale bar 5 µm (Images from Tamada et al. (2017) J Comp Neurol) (TIF 25514 KB)

## Data Availability

All the relevant data used in this study can be accessed upon reasonable request from the corresponding author.
