# Supplementary figures and images for: Three-dimensional ultrastructure analysis of organelles in injured motor neuron
Source: Anat Sci Int. 2023 Apr 18;98(3):360–9. doi: 10.1007/s12565-023-00720-y (PMC10256651; doi:10.1007/s12565-023-00720-y)

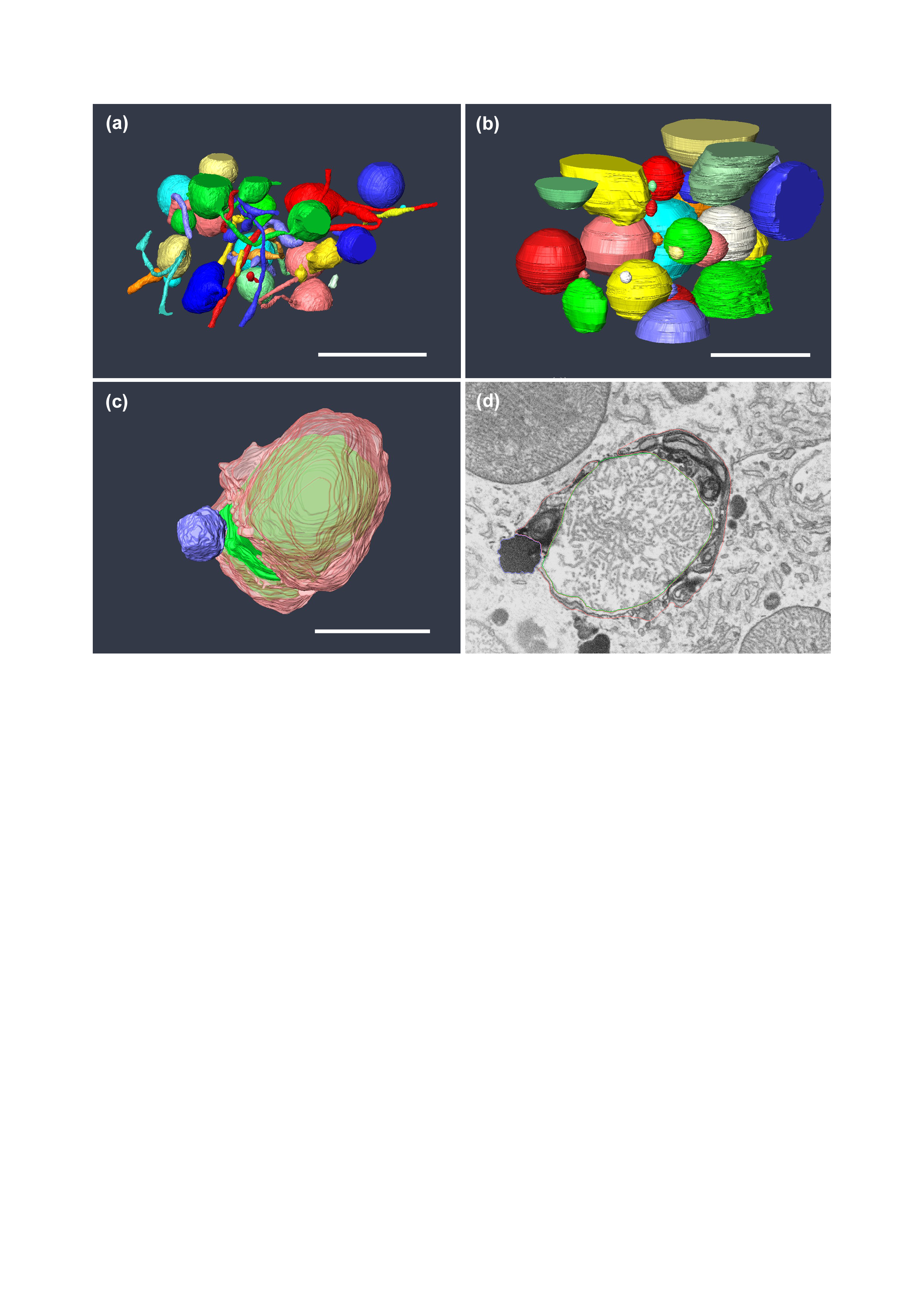

Supplement: Supplementary file 1 — FIB/SEM analysis of mitochondrial in injured neurons of Drp1 KO mouse (a) Each mitochondrion in soma of injured motor neurons was reconstructed in different colours. At one week after injury, some swollen mitochondria with processes are observed. (b) At two weeks after injury, extremely large round mitochondria without processes are detected. (c) Mitophagy-like structures with lysosomes are observed at two weeks after injury (green: mitochondria, pink: isolation membranes, purple: lysosome). (d) The representative SEM image for (c). The inner structures of mitochondria are also collapsed. Scale bar 5 µm (Images from Tamada et al. (2017) J Comp Neurol) (TIF 25514 KB) [file 12565_2023_720_MOESM1_ESM.tif]
